# Effect of Transition Metal Substitution on the Structure and Properties of a Clathrate-Like Compound Eu_7_Cu_44_As_23_

**DOI:** 10.3390/ma9070587

**Published:** 2016-07-19

**Authors:** Igor V. Plokhikh, Dmitri O. Charkin, Valeriy Yu. Verchenko, Ivan A. Ignatyev, Sergey M. Kazakov, Alexey V. Sobolev, Igor A. Presniakov, Alexander A. Tsirlin, Andrei V. Shevelkov

**Affiliations:** 1 Department of Chemistry, Lomonosov Moscow State University, Moscow 119991, Russia; ig.plohih@yandex.ru (I.V.P.); charkin@inorg.chem.msu.ru (D.O.C.); verchenko@inorg.chem.msu.ru (V.Y.V.); ivan-ignatyev1997@yandex.ru (I.A.I.); kazakov@icr.chem.msu.ru (S.M.K.); sobolev@radio.chem.msu.ru (A.V.S.); ipresniakov@rambler.ru (I.A.P.); 2 National Institute of Chemical Physics and Biophysics, Tallinn 12618, Estonia; 3 Experimental Physics VI, Center for Correlations and Magnetism, Institute of Physics, University of Augsburg, Augsburg 86135, Germany; altsirlin@gmail.com

**Keywords:** clathrate, synthesis, crystal structure, magnetic properties, energy production, conversion, storage

## Abstract

A series of substitutional solid solutions—Eu_7_Cu_44−*x*_T*_x_*As_23_ (T = Fe, Co, Ni)—based on a recently discovered clathrate-like compound (Eu_7_Cu_44_As_23_) were synthesized from the elements at 800 °C. Almost up to 50% of Cu can be substituted by Ni, resulting in a linear decrease of the cubic unit cell parameter from *a* = 16.6707(1) Å for the ternary compound to *a* = 16.3719(1) Å for the sample with the nominal composition Eu_7_Cu_24_Ni_20_As_23_. In contrast, Co and Fe can only substitute less than 20% of Cu. Crystal structures of six samples of different composition were refined from powder diffraction data. Despite very small differences in scattering powers of Cu, Ni, Co, and Fe, we were able to propose a reasonable model of dopant distribution over copper sites based on the trends in interatomic distances as well as on Mössbauer spectra for the iron-substituted compound Eu_7_Cu_36_Fe_8_As_23_. Ni doping increases the Curie temperature to 25 K with respect to the parent compound, which is ferromagnetically ordered below 17.5 K, whereas Fe doping suppresses the ferromagnetic ordering in the Eu sublattice.

## 1. Introduction

Clathrates belong to a peculiar class of inclusion compounds with a complete segregation of guests inside large polyhedral cages forming a framework. More than 250 inorganic/intermetallic clathrate compounds are known. They are grouped into 10 structure types and include over 40 chemical elements as constituents of host and guest substructures [1]. Despite such a structural and chemical variety, the nature of guests is typically limited to alkali and alkali-earth metals for anionic clathrates, and to halogens and chalcogens for inverse clathrates. However, there are several compounds that feature rare-earth elements Ce, Pr, or Eu. These examples are relatively scarce and feature type-I and type-VIII clathrates only. Nevertheless, the presence of a rare-earth cation gives rise to various interesting properties, including the enhancement of thermopower [2] and the formation of magnetic order [3,4] that can trigger a giant magnetocaloric effect [5,6].

**Figure 1:**
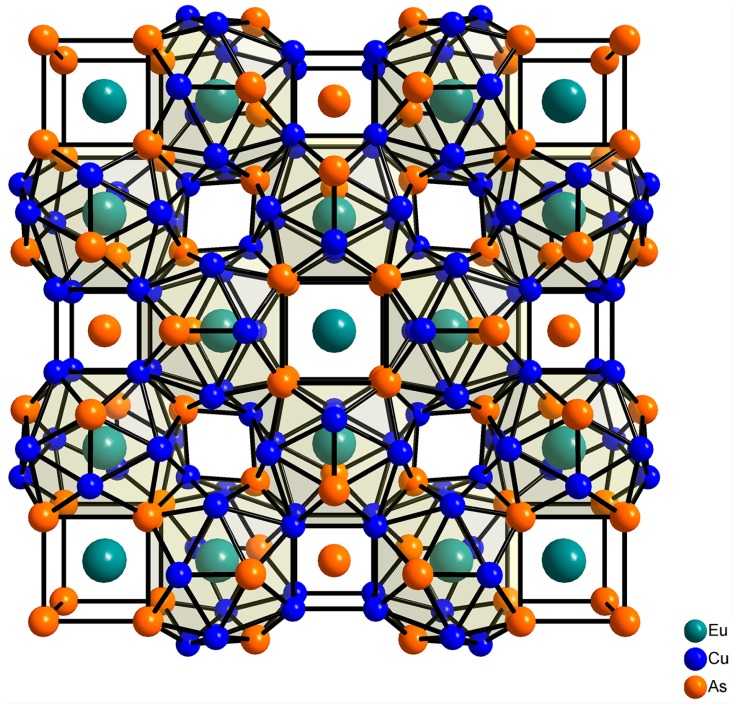
Projection of the Eu_7_Cu_44_As_23_ crystal structure onto the (001) plane.

A combination of europium, copper, and a group 15 metals gives rise to a broad family of ternary compounds with a great variety of crystal structures and properties. The structures range from pseudo-layered—related to the types known for Fe–As based superconductors [7,8]—to truly three-dimensional structures, in which europium occupies large voids and displays high coordination numbers. The vast majority of these compounds contain divalent europium, with the 4*f*^7^ ground state configuration giving rise to strong paramagnetic response and, eventually, magnetic ordering. It is worth noting that the pseudo-layered compounds typically feature antiferromagnetic (AFM) ordering [7], whereas ferromagnetic (FM) ordering is rare—Eu_2_Cu_6_P_5_ and EuCu_4_P_3_ being the only examples [9]. On the contrary, compounds with 3D structures frequently display FM ordering, as in the clathrate compound Eu*_x_*Ba_8−*x*_Cu_16_P_30_, exhibiting a superstructure of the type-I clathrate [10], and in the recently discovered clathrate-like compound Eu_7_Cu_44_As_23_ [11]. Whereas the former is a typical clathrate compound whose structure and properties can be rationalized using the Zintl–Klemm approach, the latter phase is an unbalanced metallic compound. Its resemblance to clathrates is ensured by a high coordination number of the Eu^2+^cations. In its crystal structure (Figure 1), two types of Eu^2+^cations alternate, one of which resides in a cubic environment of eight arsenic atoms, whereas the other one sits in the center of a 20-vertex polyhedral void. Low-temperature thermodynamic measurements revealed ferromagnetic behavior of Eu_7_Cu_44_As_23_ below 17.5 K, owing to the interaction between the localized 4*f*^7^ Eu^2+^cations, presumably through the conducting Cu–As framework.

Given the electronic imbalance and the corresponding metallic behavior of Eu_7_Cu_44_As_23_, we considered that extended solid solutions could be formed by substituting Cu with its neighboring 3*d*-elements possessing lesser number of valence electrons than copper. In this paper, we present synthesis and the investigation of solid solutions formed by substituting Fe, Co, or Ni for copper in Eu_7_Cu_44_As_23_, and discuss the influence of such substitutions on structural and magnetic properties.

## 2. Results

### 2.1. Synthesis and Homogeneity Ranges

**Figure 2:**
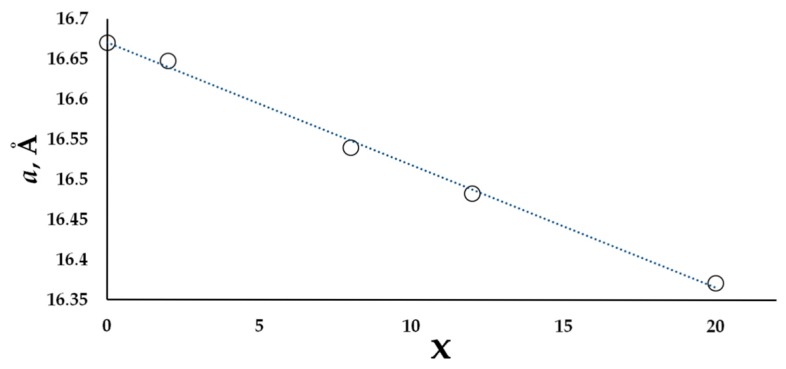
Cubic unit cell parameters vs. *x* in solid solutions Eu_7_Cu_44−*x*_Ni*_x_*As_23_. Markers cover standard deviations.

An optimal synthetic procedure for the solid solutions is almost the same as for the parent compound. The only difference is that we had to increase the annealing time by 48 h in order to reach equilibrium. The largest homogeneity range was observed for T = Ni, with an almost linear (Figure 2) decrease of the unit cell parameter from *a* = 16.6707(2) Å [11] for the undoped phase to *a* = 16.3719(1) Å for the sample with *x* = 20 (the sample with the nominal composition Eu_7_Cu_24_Ni_20_As_23_ contains up to 5% EuNi_5_As_3_). The homogeneity range for Co and Fe were found to be narrower, with the substitution limit of *x* = 8.

### 2.2. Crystal Structure Refinement and Description

Refinement of powder XRD samples with the crystal structure of Eu_7_Cu_44_As_23_ as a starting model resulted in low residuals for all samples (Table 1 and Table 2). This fact indicates that no significant structure distortion occurs during the substitution. In all cases, we observe a gradual decrease of the cubic cell parameter with almost the same increment (about 0.1% per atom) for T = Co and Ni, while for T = Fe, the increment is smaller, about 0.04% per atom.

**Figure 3:**
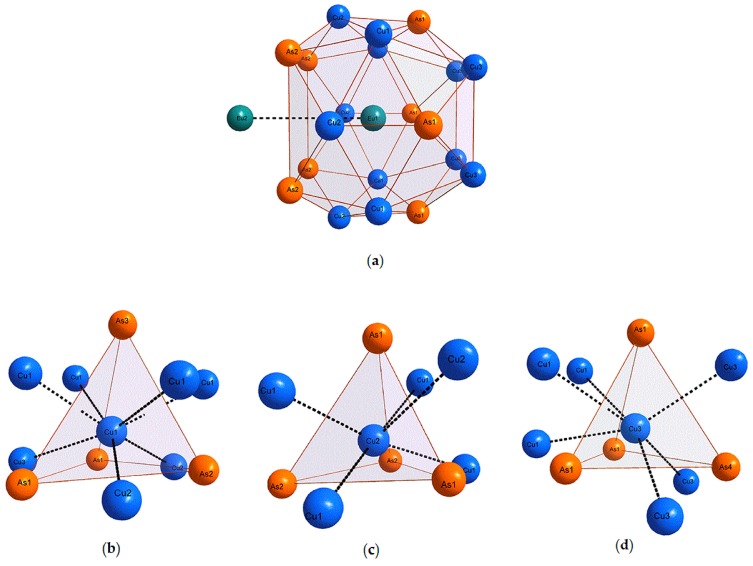
(**a**–**d**) Coordination polyhedra of Eu1, Cu1, Cu2, and Cu3, respectively, in the crystal structure of Eu_7_Cu_44_As_23_.

**Figure 4:**
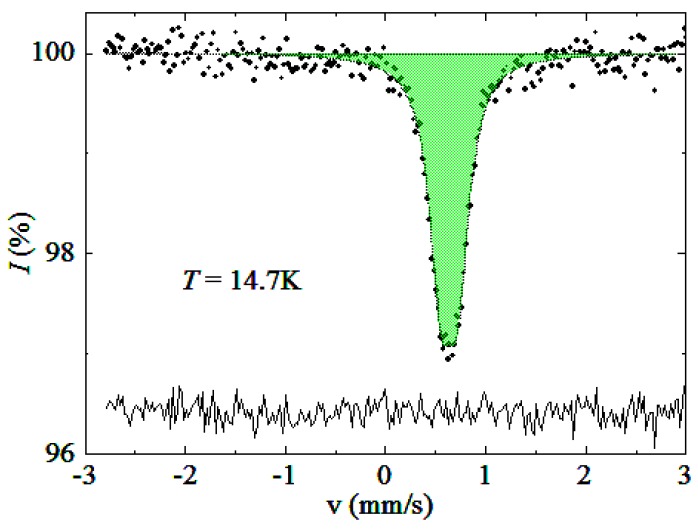
^57^Fe Mössbauer spectrum of Eu_7_Cu_36_Fe_8_As_23_ recorder at 15 K.

A general view of the crystal structure of Eu_7_Cu_44−*x*_T*_x_*As_23_ is shown in Figure 1. The 3D framework built of As and T features large voids occupied by Eu, having a 20-fold coordination composed of eight As + 12T atoms (Figure 3a), with the distances to the neighbors varying from 3.13 to 3.54 Å. The remaining Eu atoms occupy smaller cubic voids formed solely by the As atoms. Within the framework, the T and As atoms occupy three and four crystallographic sites, respectively. The coordination of the framework atoms is quite different; importantly, there are no As-As bonds, which suggests that all As atoms can be considered as As^−3^ anions. Additionally, each of three independent T atoms has four As neighbors forming slightly distorted tetrahedra, and the coordination is supplemented by five, six, or seven T atoms (Figure 4b–d). In general, the atomic arrangement resembles that found in the crystal structure of BaHg_11_ [13], with the doubling of a cubic unit cell parameter, *a*(Eu_7_Cu_44−*x*_T*_x_*As_23_) ≈ 2*a*(BaHg_11_).

### 2.3. Mössbauer Spectra

Our X-ray diffraction data did not allow us to distinguish between Cu and the substituting T element. Therefore, we used other methods in order to shed more light onto the distribution of the T atoms. To this end, we chose the sample with the Eu_7_Cu_36_Fe_8_As_23_ composition and performed the ^57^Fe-Mössbauer study at low temperatures. The experimental spectrum presented in Figure 4 consists of a single narrow quadrupole doublet with the hyperfine parameters listed in Table 3. We note that these parameters are not sensitive to temperature in the entire investigated range. The isomer shift of 0.63–0.64 mm·s^−1^ and quadrupole splitting of 0.17–0.18 mm·s^−1^ are characteristic of high-spin Fe^3+^cations in a symmetric coordination environment with a high coordination number. We note, however, that the observed value of the isomer shift is higher than those reported for other compounds exhibiting numerous Fe–Fe bonds. For instance, the isomer shift of 0.30–0.43 mm·s^−1^ was found for Fe_3_GeTe_2_ [14], where the coordination number of iron ranges from eight to ten, including up to four Fe–Fe bonds. We believe that the difference might arise from shorter Fe–As bonds in our compound compared to Fe–Ge and Fe–Te bonds in Fe_3_GeTe_2_.

The constant hyperfine parameters in the temperature range of 14.7–41.1 K rule out the possibility of electron transfer between the transition metal atoms, whereas a very low half-width of the doublet points at a single coordination site occupied by the iron atoms. This is because the difference in the coordination numbers of Fe in the T1, T2, and T3 positions would result in essentially dissimilar hyperfine parameters. However, as long as three metal sites possess similar—though not identical—coordination, the ^57^Mössbauer spectrum alone cannot distinguish which site is preferred by Fe in the crystal structure of Eu_7_Cu_36_Fe_8_As_23_.

### 2.4. Interatomic Distances

Substitution of Ni for Cu provides the most extended solid solution up to *x_max_* = 20. Upon substitution, the cubic unit cell parameter decreases almost linearly with the composition (Figure 2). The same trend is observed for the majority of bond distances (Table 4, Figure 3).

**Figure 5:**
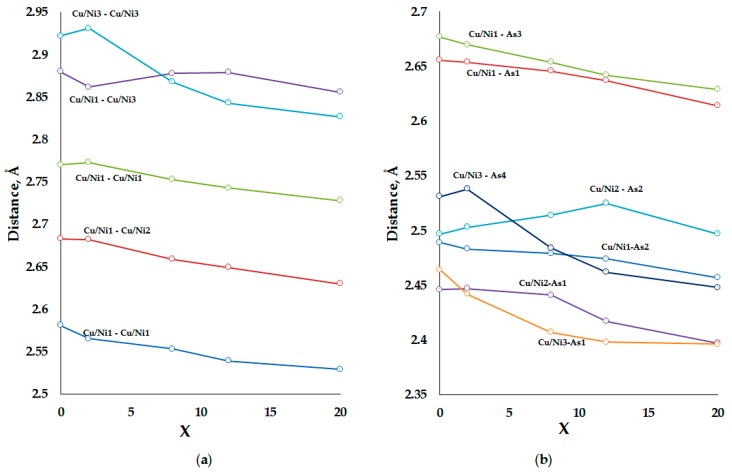
The (**a**) Cu/Ni–As; and (**b**) Cu/Ni–Cu/Ni distances in the structure of Eu_7_Cu_44−*x*_Ni*_x_*As_23_ for different *x*. Cu/Ni denotes mixed sites of Cu and Ni. The standard deviations are below the sizes of experimental datapoints. The lines are drawn to guide the eye.

However, exceptions are present, most importantly within the [TAs_4_] tetrahedra. Figure 5a shows that the T3–As1 and T3–As4 distances display the greatest shrinking upon the substitution, decreasing by 0.07 and 0.08 Å, respectively (Table 3). This is in striking contrast with the minor changes in the T1–As bonding distances as well as the T2–As distances, the latter indicating that the T2As_4_ tetrahedra distort rather than exhibit a linear decrease in the bonding distances. Consequently, it can be assumed that Ni—having a smaller covalent radius than Cu—prefers the T3 position until it is fully filled at *x* = 8 (32*f* site, *Z* = 4), after which Ni starts to occupy other positions. This is further corroborated by the T-T distances presented in Figure 5b, which shows that the T3–T3 intermetallic distance displays the greatest decrease upon the Ni-for-Cu substitution. The Fe-for-Cu substitution stops at *x* = 8, and we do not have enough data for the similar analysis of interatomic distances. However, we note that only the T3–As and T3–T3 interatomic distances decrease substantially. Taking into account that the Mössbauer data points at a single position of the iron atoms, we believe that Fe most likely occupies the T3 site (Table 3).

### 2.5. Magnetic Properties

**Figure 6:**
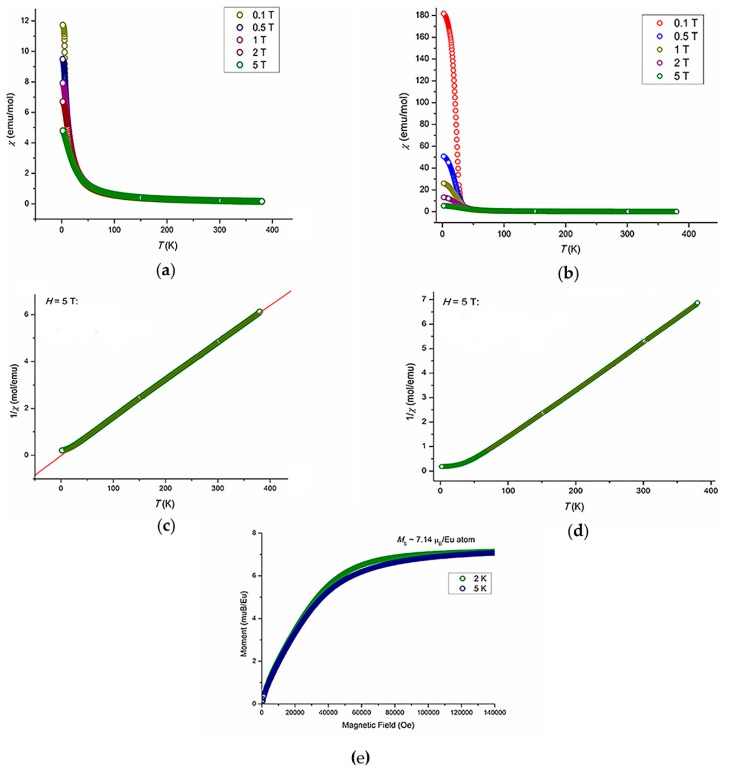
Magnetic susceptibility vs. temperature for (**a**) Eu_7_Cu_36_Fe_8_As_23_ and (**b**) Eu_7_Cu_42_Ni_2_As_2_; inverse magnetic susceptibility vs. temperature for (**c**) Eu_7_Cu_36_Fe_8_As_23_ and (**d**) Eu_7_Cu_42_Ni_2_As_2_; (**e**) magnetization vs. field for Eu_7_Cu_36_Fe_8_As_23_.

Temperature dependences of the magnetic susceptibility for Eu_7_Cu_36_Fe_8_As_23_and Eu_7_Cu_42_Ni_2_As_23_ are presented in Figure 6a,b respectively.

The former sample behaves as a typical Curie–Weiss paramagnet (Figure 6c), with the Weiss temperature only slightly exceeding zero, θ*_W_* = 0.95 K. A deviation from the Curie–Weiss behavior at low temperatures with a visible increase of the magnetic susceptibility likely stems from minor paramagnetic impurities. The calculated magnetic moment *M*_eff_ = 8.32 μ_B_ per Eu-atom is noticeably higher than the expected value for pure Eu^2+^ (*M_eff_* = 7.94 μ_B_ for *J* = *S* = 7/2), indicating that some contribution from iron is also present. Assuming additivity of the magnetic moments, where a square of the effective moment is a sum of the squares of individual moments, we obtain

(1) $$\mu_{eff}\left(Fe\right)=\sqrt{8.32^{2}-7.94^{2}}\times \frac{7}{8}=2.18\mu_{B}$$

per one Fe-atom, which is in the typical range for Fe-based itinerant magnets (compare to 2 μ_B_ in FeSi [15]). Note that the paramagnetic effective moments are calculated as μ_eff_ = *g*[*S*(*S* + 1)]^1/2^μ_B_ and, for example, μ_eff_ of Eu^2+^ is 7.94 μ_B_ assuming *g* = 2.0 for *J* = 0 (4*f*^14^). On the other hand, Fe only weakly contributes to the ordered moment (Figure 6e) at low temperatures,

(2) $$\mu_{ord}\left(Fe\right)=\left(7.14-7.0\right)\times \frac{7}{8}=0.123\mu_{B}$$

The presence of the magnetic contribution from Fe atoms is also consistent with their high-spin state inferred from the Mössbauer spectra.

In contrast to Eu_7_Cu_36_Fe_8_As_23_, Eu_7_Cu_42_Ni_2_As_23_ exhibits FM ordering below 25 K, whereas above *T_C_* it behaves as a Curie–Weiss paramagnet (Figure 6d) with the effective moment of 7.89 μ_B_, which is only slightly lower than the expected value of 7.94 for Eu^2+^ (4*f*^7^). The Curie–Weiss temperature extracted (Figure 6d) from the high-temperature paramagnetic susceptibility (θ*_W_* = 25 K) coincides with *T*_C_, showing that the FM ordering stems from localized Eu^2+^cations. It is worth noting that the parent compound Eu_7_Cu_44_As_23_ orders ferromagnetically at 17.5 K; above this temperature, it behaves as a Curie–Weiss paramagnet with the effective moment of 7.94 μ_B_ and at 2 K the moment saturates with the saturation moment *M*_S_ = 7.0 μ_B_. These observations suggest that the localized Eu^2+^ (4*f*^7^) cations undergo FM ordering. Taking into account that the shortest Eu-Eu separation exceeds 4 Å and the compound displays metallic conductivity, one can assume that the Eu^2+^cations interact through the conduction electrons of the Cu–As clathrate-like framework. The partial substitution of iron for copper in the framework drastically changes the magnetic properties. The FM ordering is lost, and, as long as the Eu–Eu separation does not change substantially (by 0.08 Å only), we believe that the change in the charge carrier concentration within the framework is responsible for the modification of magnetic properties upon doping. In the case of Eu_7_Cu_42_Ni_2_As_23_, a different substitution picture seems to appear because Ni tends to behave as an effectively d^10^ atom in many intermetallic and related compounds [16,17]. As a result, it shows no magnetic moment that could interfere the FM ordering of Eu^2+^cations, which is observed at 25 K.

## 3. Discussion

Recently we have discovered two new arsenides, namely Eu_7_Cu_44_As_23_ and Sr_7_Cu_44_As_23_, which are the first representatives of a new structure type derived from the intermetallic compound BaHg_11_. As often observed for ternary arsenides of coinage and alkaline earth metals, these two compounds are the only representatives showing that the new crystal structure is sensitive to the radius of the A-cation. This is not surprising as long as the crystal structure is quite complex and demonstrates a clathrate-like environment of 6/7 of Eu(Sr) atoms by 20 distant Cu and As atoms, whereas the rest of the Eu(Sr) atoms reside in the cubic voids built of eight copper atoms. Such a combination of structure elements requires precise matching of atomic sizes. As a result, isostructural compounds with smaller Ca or larger Ba do not form due to an apparent size mismatch.

Since Eu_7_Cu_44_As_23_ is electronically unbalanced and demonstrates a metallic type of conductivity, we supposed that extended solid solutions could be formed by substituting copper with 3*d*-elements having lower number of valence electrons but similar atomic radius. Indeed, our assumption proved to be correct and we have observed homogeneity ranges of Eu_7_Cu_44−*x*_T*_x_*As_23_ (T = Fe, Co, Ni) extended to 50% for Ni and 20% in the cases of Co and Fe. A close location of Cu and the substituting element T in the Periodic Table resulted in a quite challenging task of determining the dopants distribution among copper sites. The Rietveld refinement against powder X-ray diffraction data was not sensitive enough (as expected); nevertheless, it allowed us to analyze changes in interatomic distances with T for Cu substitution and to indicate the most probable position for the T for Cu substitution. This assumption was facilitated by the ^57^Fe Mössbauer data for the iron-containing sample, which confirmed that Fe substitutes for Cu only at a single site. Interestingly, the substitution of Cu by Fe leads to suppression of ferromagnetic ordering in Eu-sublattice, while small amounts of Ni increase T_C_ with respect to the parent phase.

The obtained results call for further investigation aimed at expanding our knowledge about this structure type. The main challenge is to rationalize the FM-ordering mechanism in Eu_7_Cu_44_As_23_ and to check for possible magnetocaloric effect near the transition temperature. Another important task is to examine the geometrical and electronic limits for the Eu_7_Cu_44_As_23_ structure type by partially substituting Eu by Na, Ca, or Ce, and As by Sb, Ge, and Te. The respective research is currently in progress.

## 4. Materials and Methods

### 4.1. Synthesis and Primary Characterization

The starting materials were ingots of Eu, Cu, Fe, Co, and Ni, as well as As powder of at least analytical grade. The procedure was essentially the same as for the previously reported A_7_Cu_44_As_23_ (A = Eu, Sr) [11]. Prior to use, the Fe, Co, and Ni powders were annealed in hydrogen to remove surface oxide. All operations were performed in an Ar-filled glovebox (M’Braun, p(O_2_, H_2_O) < 1 ppm). The elements were mixed according to the composition Eu_7_Cu_44−*x*_T*_x_*As_23_, pressed into pellets, loaded in carbon-lined silica tubes, evacuated to ~0.05 mTorr and annealed at 200, 400, 600, and 800 °C (ramp 1 °C/min, soak 12 h). The obtained samples were ground, pressed, and annealed at 800 °C for 48 h, three times. The phase composition was checked using a Bruker D8/Advance diffractometer (CuKα_1,2_ radiation, LynxEye PSD).

### 4.2. Crystal Structure Determination

**Figure 7:**
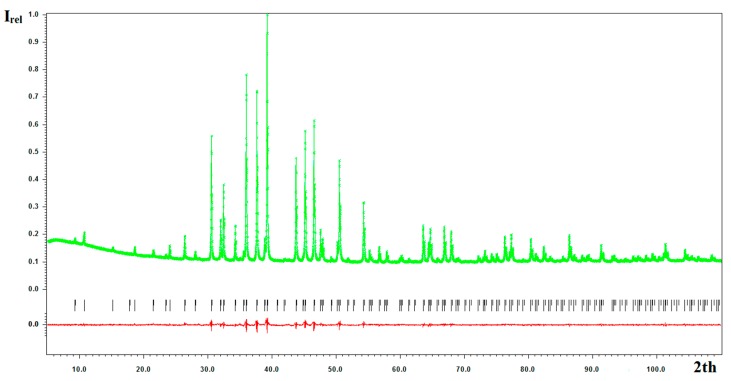
The Rietveld refinement plot for Eu_7_Cu_36_Ni_8_As_23_. Experimental profile, green; peak positions, black; differential profile, red.

Phase-pure or nearly phase-pure powder samples were used for the crystal structure refinement. PXRD data were collected on Powder X’Pert diffractometer (CuKα_1,2_ radiation, PANalytical, Almelo, The Netherlands) and processed using the Jana2006 package [12] utilizing the Rietveld method with the crystal structure of Eu_7_Cu_44_As_23_ as a starting model. At the first step, profile parameters and atomic coordinates were refined, while atomic displacement parameters for all atoms were fixed at the value of 0.01 Å^2^, and the distribution of T (T = Fe, Co, Ni) atoms over three copper sites was set to be random. The attempt to refine the atomic displacement of all atoms simultaneously led to unrealistic values for the As atom (close to zero or negative); consequently, they were fixed, and the T/Cu ratio (T = Fe, Co, Ni) was refined at the Cu sites. Then, this ratio was fixed, and atomic displacement parameters were refined. When the satisfactory values of atomic displacement parameters were obtained, we checked the occupancy of Eu and As atoms—which appeared to be close to unity—and were then fixed at their ideal values. Details of the refinement, refined structural parameters, and selected interatomic distances are collected in Table 1, Table 2 and Table 3, respectively. A typical Rietveld plot is presented in Figure 7.

### 4.3. Magnetic Properties

Magnetic susceptibility measurements were carried out with the vibrating sample magnetometer (VSM) setup of a Physical Property Measurement System (PPMS, Quantum Design, San Diego, CA, USA). The data were collected in external magnetic fields between 0 T and 14 T in the temperature range of 2–380 K.

### 4.4. Mössbauer Study

^57^Fe Mössbauer spectra of the sample implemented within the closed-cycle refrigerator system were recorded between 10 and 50 K using a conventional constant-acceleration spectrometer MS-1104Em in the transmission geometry. The radiation source ^57^Co(Rh) was kept at room temperature. All isomer shifts are referred to α-Fe at 300 K. The experimental spectra were processed and analyzed using the SpectrRelax program [18].

## 5. Conclusions

In conclusion, we studied possibilities of copper substitution in the recently discovered clathrate-like compound Eu_7_Cu_44_As_23_. We showed that up to nearly 50% of Cu can be substituted by Ni, and almost 20% can be substituted by Fe and Co. Based on the X-ray structure analysis and Mössbauer spectroscopy, we analyzed the distribution of dopants among Cu sites. We showed that the introduction of even a small amount of Ni increases T_С_, while Fe doping suppresses ferromagnetic ordering in the Eu-sublattice.

## Figures and Tables

**Table 1:** Details of the powder XRD experiment for Eu_7_Cu_44−*x*_T*_x_*As_23_ phases (space group) ^2^.

Composition (T, *x*)	Ni, 2	Ni, 8	Ni, 12	Ni, 20	Co, 8	Fe, 8
*Z* Cell parameters	4					
*a*, Å	16.6487(2)	16.5407(1)	16.4830(1)	16.3719(1)	16.5421(1)	16.6251(1)
*V*, Å^3^	4614.65(8)	4525.42(3)	4478.25(3)	4388.34(6)	4526.57(3)	4595.11(1)
Calculated density, g/cm^3^	8.0359	8.1253	8.1451	8.2506	8.1599	7.9947
Radiation	CuKα_1,2_					
2θ range	5–140	5–110	5–110	5–120	5–110	5–110
Data points/reflections	10,282/271	7996/186	7996/184	8758/205	7996/186	1997/187
Overall/structural parameters	44/17	43/17	52/17	62/17	56/17	46/20
Analyzing package *R* values ^1^ (%):	Jana 2006 [12]					
*R_B_*	3.60	1.92	2.03	1.62	1.94	1.76
*R_p_*	1.32	1.13	1.51	1.15	0.81	1.35
*R_exp_*	0.81	0.74	1.44	1.13	0.55	1.23
*GOF*	2.39	2.26	1.38	1.30	2.47	1.47

^1^ *R_B_*—Bragg *R*-factor; *R_P_*—profile *R*-factor; ^2^ Further details of the crystal structures can be found in Supplementary Materials.

**Table 2:** Refined atomic parameters for Eu_7_Cu_44−*x*_T*_x_*As_23_.

**T = Ni, *x* = 2**					
**Atom**	**Position**	***x*/*a***	***y*/*b***	***z*/*c***	***U_iso_***
Eu1	*24e*(*x*, 0, 0)	0.2447(1)	-	-	0.0101(5)
Eu2	*4a*(0, 0, 0)	-	-	-	0.005(1)
Cu/Ni1	*96k*(*x*, *x*, *z*)	0.13666(4)	-	0.2544(1)	0.0168(6)
Cu/Ni2	*48h*(0, *y*, *y*)	-	0.1825(1)	-	0.0161(9)
Cu/Ni3	*32f*(*x*, *x*, *x*)	0.41199(9)	-	-	0.021(1)
As1	*48i*(½, *y*, *y*)	-	0.17097(8)	-	0.0080(6)
As2	*32f*(*x*, *x*, x)	0.11008(7)	-	-	0.0072(7)
As3	*8c*(¼, ¼, ¼)	-	-	-	0.0034(9)
As4	*4b*(½, ½, ½)	-	-	-	0.042(3)
**T = Ni, *x* = 8**					
**Atom**	**Position**	***x*/*a***	***y*/*b***	***z*/*c***	***U_iso_***
Eu1	*24e*(*x*, 0, 0)	0.24356(9)	-	-	0.0066(3)
Eu2	*4a*(0, 0, 0)	-	-	-	0.0024(9)
Cu/Ni1	*96k*(*x*, *x*, *z*)	0.13658(4)	-	0.2543(1)	0.0105(3)
Cu/Ni2	*48h*(0, *y*, *y*)	-	0.18399(7)	-	0.0124(6)
Cu/Ni3	*32f*(*x*, *x*, *x*)	0.41328(8)	-	-	0.0121(8)
As1	*48i*(½, *y*, *y*)	-	0.16919(6)	-	0.0072(4)
As2	*32f*(*x*, *x*, *x*)	0.10942(5)	-	-	0.0085(5)
As3	*8c*(¼, ¼, ¼)	-	-	-	0.0087(7)
As4	*4b*(½, ½, ½)	-	-	-	0.048(2)
**T = Ni, *x* = 12**					
**Atom**	**Position**	***x*/*a***	***y*/*b***	***z*/*c***	***U_iso_***
Eu1	*24e*(*x*, 0, 0)	0.2434(1)	-	-	0.0054(4)
Eu2	*4a*(0, 0, 0)	-	-	-	0.002(1)
Cu/Ni1	*96k*(*x*, *x*, *z*)	0.13670(5)	-	0.2544(1)	0.0083(4)
Cu/Ni2	*48h*(0, *y*, *y*)	-	0.18523(9)	-	0.0104(8)
Cu/Ni3	*32f*(*x*, *x*, *x*)	0.4138(1)	-	-	0.0095(10)
As1	*48i*(½, *y*, *y*)	-	0.16905(7)	-	0.0039(5)
As2	*32f*(*x*, *x*, *x*)	0.10934(7)	-	-	0.0066(7)
As3	*8c*(¼, ¼, ¼)	-	-	-	0.0023(9)
As4	*4b*(½, ½, ½)	-	-	-	0.020(2)
**T = Ni, *x* = 20**					
**Atom**	**Position**	***x*/*a***	***y*/*b***	***z*/*c***	***U_iso_***
Eu1	*24e*(*x*, 0, 0)	0.2425(1)	-	-	0.0032(3)
Eu2	*4a*(0, 0, 0)	-	-	-	0.009(1)
Cu/Ni1	*96k*(*x*, *x*, *z*)	0.13647(3)	-	0.2543(1)	0.0047(3)
Cu/Ni2	*48h*(0, *y*, *y*)	-	0.18447(8)	-	0.0016(7)
Cu/Ni3	*32f*(*x*, *x*, *x*)	0.41368(8)	-	-	0.0073(9)
As1	*48i*(½, *y*, *y*)	-	0.16986(6)	-	0.0035(4)
As2	*32f*(*x*, *x*, *x*)	0.10923(5)	-	-	0.0042(5)
As3	*8c*(¼, ¼, ¼)	-	-	-	0.0016(7)
As4	*4b*(½, ½, ½)	-	-	-	0.004(2)
**T = Co, *x* = 8**					
**Atom**	**Position**	***x*/*a***	***y*/*b***	***z*/*c***	***U_iso_***
Eu1	*24e*(*x*, 0, 0)	0.24150(9)	-	-	0.0094(3)
Eu2	*4a*(0, 0, 0)	-	-	-	0.010(1)
Cu/Co1	*96k*(*x*, *x*, *z*)	0.13653(4)	-	0.25467(1)	0.0161(8)
Cu/Co2	*48h*(0, *y*, *y*)	-	0.18542(8)	-	0.018(7)
Cu/Co3	*32f*(*x*, *x*, *x*)	0.41383(8)	-	-	0.014(5)
As1	*48i*(½, *y*, *y*)	-	0.16785(6)	-	0.0110(5)
As2	*32f*(*x*, *x*, *x*)	0.10955(6)	-	-	0.0122(6)
As3	*8c*(¼, ¼, ¼)	-	-	-	0.0092(8)
As4	*4b*(½, ½, ½)	-	-	-	0.023(2)
**T = Fe, *x* = 8**					
**Atom**	**Position**	***x*/*a***	***y*/*b***	***z*/*c***	***U_iso_***
Eu1	*24e*(*x*, 0, 0)	0.23912(9)	-	-	0.0088(3)
Eu2	*4a*(0, 0, 0)	-	-	-	0.010(1)
Cu/Fe1	*96k*(*x*, *x*, *z*)	0.13663(4)	-	0.2547(1)	0.0140(4)
Cu/Fe2	*48h*(0, *y*, *y*)	-	0.18436(8)	-	0.0118(7)
Cu/Fe3	*32f*(*x*, *x*, *x*)	0.41385(8)	-	-	0.0115(8)
As1	*48i*(½, *y*, *y*)	-	0.16843(6)	-	0.0074(5)
As2	*32f*(*x*, *x*, *x*)	0.11001(6)	-	-	0.0048(5)
As3	*8c*(¼, ¼, ¼)	-	-	-	0.0033(8)
As4	*4b*(½, ½, ½)	-	-	-	0.020(2)

**Table 3:** Hyperfine parameters of the ^57^Fe Mössbauer spectra of Eu_7_Cu_36_Fe_8_As_23_ at different temperatures; δ is the isomer shift, Δ is the quadrupole splitting, and *W* is the linewidth.

*T*, K (±1 K)	δ (mm/s)	∆ (mm/s)	*W* (mm/s)
15	0.636(3)	0.169(7)	0.28(2)
27	0.638(3)	0.176(6)	0.27(2)
41	0.633(2)	0.166(7)	0.29(2)

**Table 4:** Selected interatomic distances (in Å) for Eu_7_Cu_44−*x*_T*_x_*As_23_. Cu/T denotes a mixed site of Cu and T (T = Fe, Co, Ni).

Composition (T, *x*)		Undoped	Ni, 2	Ni, 8	Ni, 12	Ni, 20	Co, 8	Fe, 8
Eu1	1 × Eu2	4.0863(2)	4.074(2)	4.029(2)	4.012(2)	3.970(2)	3.993(2)	3.975(2)
	4 × Cu/T1	3.2217(3)	3.2217(7)	3.1999(6)	3.1917(8)	3.1657(6)	3.2010(8)	3.2227(7)
	4 × Cu/T2	3.2092(2)	3.210(2)	3.199(1)	3.200(2)	3.166(1)	3.203(2)	3.197(1)
	4 × Cu/T3	3.4702(2)	3.471(2)	3.464(2)	3.453(2)	3.442(2)	3.493(2)	3.541(2)
	4 × As1	3.1814(1)	3.174(2)	3.149(1)	3.138(1)	3.129(1)	3.157(1)	3.194(1)
	4 × As2	3.4326(2)	3.427(2)	3.387(1)	3.373(2)	3.340(1)	3.365(2)	3.361(1)
Eu2	8 × As2	3.1659(3)	3.174(1)	3.1346(9)	3.122(1)	3.0974(9)	3.139(1)	3.1677(9)
	6 × Eu1	4.0863(2)	4.074(2)	4.029(2)	4.012(2)	3.970(2)	3.992(2)	3.975(2)
Cu/T1	2 × Cu/T1	2.5811(5)	2.565(2)	2.553(2)	2.539(2)	2.529(2)	2.544(2)	2.556(2)
	2 × Cu/T1	2.7703(5)	2.773(2)	2.752(2)	2.743(2)	2.728(2)	2.766(2)	2.775(2)
	2 × Cu/T2	2.6830(2)	2.682(1)	2.659(1)	2.649(1)	2.630(1)	2.659(1)	2.675(1)
	1 × Cu/T3	2.8802(5)	2.862(2)	2.878(2)	2.856(2)	2.856(2)	2.881(3)	2.900(2)
	2 × As1	2.6560(2)	2.654(1)	2.646(1)	2.637(1)	2.614(1)	2.647(1)	2.660(1)
	1 × As2	2.4887(4)	2.483(2)	2.479(2)	2.474(2)	2.457(2)	2.483(2)	2.485(2)
	1 × As3	2.6765(3)	2.6696(7)	2.6540(6)	2.6420(7)	2.6295(6)	2.6563(7)	2.6667(7)
	1 × Eu1	3.2217(3)	3.2217(7)	3.1999(6)	3.1917(8)	3.1657(6)	3.2010(8)	3.2227(7)
Cu/T2	2 × As1	2.4462(3)	2.447(2)	2.441(2)	2.417(2)	2.397(2)	2.445(2)	2.462(2)
	2 × As2	2.4968(3)	2.503(2)	2.514(1)	2.525(2)	2.497(1)	2.536(2)	2.530(1)
	4 × Cu/T1	2.6830(2)	2.682(1)	2.659(1)	2.649(1)	2.630(1)	2.659(1)	2.674(1)
	1 × Eu1	3.2092(2)	3.210(2)	3.199(1)	3.200(2)	3.166(1)	3.203(2)	3.197(1)
Cu/T3	3 × As1	2.4643(3)	2.442(2)	2.404(2)	2.398(2)	2.396(2)	2.383(2)	2.407(2)
	1 × As4	2.5305(5)	2.538(1)	2.484(1)	2.462(2)	2.448(1)	2.472(2)	2.481(1)
	3 × Cu/T1	2.8802(5)	2.862(2)	2.878(2)	2.856(2)	2.856(2)	2.881(3)	2.900(2)
	3 × Cu/T3	2.9220(6)	2.931(2)	2.868(2)	2.843(2)	2.827(2)	2.854(2)	2.864(2)
	3 × Eu1	3.4702(2)	3.471(2)	3.464(2)	3.453(2)	3.442(2)	3.493(2)	3.541(2)
As1	4 × Cu/T1	2.6560(2)	2.654(1)	2.646(1)	2.637(1)	2.614(1)	2.647(1)	2.660(1)
	2 × Cu/T2	2.4462(3)	2.447(2)	2.441(2)	2.417(2)	2.397(2)	2.445(2)	2.462(2)
	2 × Cu/T3	2.4643(3)	2.442(2)	2.404(2)	2.398(2)	2.396(2)	2.383(2)	2.407(2)
	2 × Eu1	3.1814(1)	3.174(2)	3.149(1)	3.138(1)	3.129(1)	3.157(1)	3.194(1)
As2	3 × Cu/T1	2.4887(4)	2.483(2)	2.479(2)	2.474(2)	2.457(2)	2.483(2)	2.485(2)
	3 × Cu/T2	2.4968(3)	2.503(2)	2.514(1)	2.525(2)	2.497(1)	2.536(2)	2.530(1)
	3 × Eu1	3.4326(2)	3.427(2)	3.387(1)	3.373(2)	3.340(1)	3.365(2)	3.361(1)
	1 × Eu2	3.1659(3)	3.174(1)	3.1346(9)	3.122(1)	3.0974(9)	3.139(1)	3.1677(9)
As3	12 × Cu/T1	2.6765(3)	2.6696(7)	2.6540(6)	2.6420(7)	2.6295(6)	2.6563(7)	2.6667(7)
As4	8 × Cu/T3	2.5305(5)	2.538(1)	2.484(1)	2.462(2)	2.448(1)	2.472(2)	2.481(1)

## Supplementary Materials

The following are available online at www.mdpi.com/1996-1944/9/7/587/s1, 6 crystallographic data files in the cif format for the crystal structures of Eu_7_Cu_42_Ni_2_As_23_, Eu_7_Cu_36_Ni_8_As_23_, Eu_7_Cu_32_Ni_12_As_23_, Eu_7_Cu_22_Ni_20_As_23_, Eu_7_Cu_36_Co_8_As_23_, and Eu_7_Cu_36_Fe_8_As_23_.

## Abbreviations

The following abbreviations are used in this manuscript:

T	Fe, Co, Ni
FM	Ferromagnetic
AFM	Antiferromagnetic

## References

[1] Shevelkov A.V., Kovnir K. (2011). Zintl clathrates. Struct. Bond..

[2] Prokofiev A., Sidorenko A., Hradil K., Ikeda M., Svagera R., Waas M., Winkler H., Neumaier K., Paschen S. (2013). Thermopower enhancement by encapsulating cerium in clathrate cages. Nat. Mater..

[3] Pacheco V., Bentien A., Carrillo-Cabrera W., Paschen S., Steglich F., Grin Y. (2005). Relationship between composition and charge carrier concentration in Eu_8_Ga_16−x_Ge_30+x_clathrates. Phys. Rev. B.

[4] Bentien A., Pacheco V., Paschen S., Grin Y., Steglich F. (2005). Transport properties of composition tuned α- and β-Eu_8_Ga_16−*x*_Ge_30+*x*_. Phys. Rev. B.

[5] Srinath S., Gass J., Rebar D.J., Woods G.T., Srikanth H. (2006). Giant magnetocaloric effect in clathrates. J. Appl. Phys..

[6] Phan M.H., Woods G.T., Chaturvedi A., Stefanoski S., Nolas G.S., Srikanth H. (2008). Long-range ferromagnetism and giant magnetocaloric effect in type VIII Eu_8_Ga_16_Ge_30_clathrates. Appl. Phys. Lett..

[7] Munevar J., Micklitz H., Alzamora M., Argüello C., Goko T., Ning F.L. (2014). Magnetism in superconducting EuFe_2_As_1.4_P_0.6_ single crystals studied by local probes. Solid State Commun..

[8] Motomitsu E., Yanagi H., Kamiya T., Hirano M., Hosono H. (2006). Synthesis, structure and physical properties of layered semiconductors MCuFCh (M = Sr, Eu; Ch = S, Se). J. Solid State Chem..

[9] Charkin D.O., Urmanov A.V., Kazakov S.M., Batuk D., Abakumov A.M., Knöner S., Gati E., Wolf B., Lang M., Shevelkov A.V. (2012). Synthesis, crystal structure, transport, and magnetic properties of novel ternary copper phosphides, A_2_Cu_6_P_5_ (A = Sr, Eu) and EuCu_4_P_3_. Inorg. Chem..

[10] Kovnir K., Köhler U., Budnyk S., Prots Y., Baitinger M., Paschen S., Shevelkov A.V., Grin Y. (2011). Introducing a magnetic guest to a tetrel-free clathrate: Synthesis, structure, and properties of Eu_x_Ba_8–x_Cu_16_P_30_ (0 ≤ x ≤ 1.5). Inorg. Chem..

[11] Charkin D.O., Demchyna R., Prots Y., Borrmann H., Burkhardt U., Schwarz U., Schnelle W., Plokhikh I.V., Kazakov S.M., Abakumov A.M. (2014). Two new arsenides, Eu_7_Cu_44_As_23_ and Sr_7_Cu_44_As_23_, with a new filled variety of the BaHg_11_ structure. Inorg. Chem..

[12] Petříček V., Dušek M., Palatinus L. (2014). Crystallographic Computing System JANA2006: General features. Z. Kristallogr..

[13] Peyronel G. (1952). Struttura della fase BaHg_11_. Gazz. Chim. Ital..

[14] Verchenko V.Y., Tsirlin A.A., Sobolev A.V., Presniakov I.A., Shevelkov A.V. (2015). Ferromagnetic order, strong magnetocrystalline anisotropy and magnetocaloric effect in the layered telluride Fe_3−δ_GeTe_2_. Inorg. Chem..

[15] Takagi S., Yasuoka H., Ogawa S., Wernick J.H. (1981). ^29^Si NMR studies of an “unusual” paramagnet FeSi–Anderson localized state model. J. Phys. Soc. Jpn..

[16] Litvinenko O.N., Kuznetsov A.N., Olenev A.V., Popovkin B.A. (2007). New mixed tellurides of nickel and Group 13–14 metals Ni_3−δ_MTe_2_ (M = Sn, In, Ga). Russ. Chem. Bull..

[17] Baranov A.I., Kloo L., Olenev A.V., Popovkin B.A., Romanenko A.I., Shevelkov A.V. (2001). Unique metallic wires in a novel quasi-1D compound: Synthesis, crystal and electronic structure, and properties of Ni_8_Bi_8_SI. J. Am. Chem. Soc..

[18] Matsnev M.E., Rusakov V.S. (2012). SpectrRelax: An application for Mössbauer spectra modeling and fitting. AIP Conf. Proc..

